# Bidirectional association between cognitive function and depressive symptoms in UK community elderly based on physical activity as mediator

**DOI:** 10.1371/journal.pone.0353689

**Published:** 2026-07-21

**Authors:** Jiahui Liu, Yalong Li, Lei Song, Mu He, Jia Liu, Yuqi Lin, Jian Ma, Haoxuan Yang, Tingting Sun, Jizhao Li

**Affiliations:** 1 Baoding University, Hebei, China; 2 School of Economics and Management, North China Electric Power University, Beijing, China; 3 School of Leisure Sports and Tourism, Beijing Sport University, Beijing, China; 4 College of Physical Education and Sports, Beijing Normal University, Beijing, China; 5 Newcastle Business School, Newcastle University, Newcastle, United Kingdom; 6 Key Laboratory of Exercise and Physical Fitness, Ministry of Education, Beijing Sport University, Beijing, China; 7 School of Martial Art and Traditional Chinese Sport, Beijing Sport University, Beijing, China; University of Hafr Al-Batin, SAUDI ARABIA

## Abstract

**Introduction:**

Previous studies have shown that physical activity (PA) can improve cognitive function (CF) and depressive symptoms in middle-aged and older adults, and that there is a bidirectional relationship between the two. However, there is a lack of research on the temporal sequence and potential mechanisms of these three factors. This study conducted a large-scale longitudinal study to explore the mediating role of PA in the bidirectional association between CF and depressive symptoms in middle-aged and older adults.

**Methods:**

A longitudinal study was conducted using data from the English Longitudinal Study of Aging (ELSA) collected in 2014−2015 (T1), 2016−2017 (T2), and 2018−2019 (T3). PA, CF, and depressive symptoms were assessed via questionnaires, with Spearman’s correlation analysis used to examine variable correlations. A Cross-Lagged Panel Model (CLPM) was constructed to test the longitudinal causal relationships among the three variables, and a longitudinal mediation model was developed to analyze the mediating role of PA.

**Results:**

A total of 6,787 participants aged 50 years or older were included, comprising 3,777 women (55.7%) and 3,010 men (44.3%), with a median baseline age of 66 years. Spearman correlation analysis indicated a significant correlation among the three variables (r_s_=[−0.225, 0.447], P < 0.001). CLPM results showed that poorer CF predicted more severe depressive symptoms in the future (β_T1-T2_ = −0.038, P < 0.001; β_T2-T3_ = −0.040, P < 0.001), and more severe depressive symptoms predicted poorer future CF (β_T1-T2_ = −0.022, P = 0.005; β_T2-T3_ = −0.021, P = 0.005). The Wald test indicated that CF was the dominant factor in the bidirectional negative relationship between CF and depressive symptoms (Wald χ² = 7.546, P = 0.006). Longitudinal mediation model results showed that poorer CF at T1 predicted lower PA at T2 (β_T1-T2_ = 0.043, P < 0.001), and lower PA at T2 predicted more severe depressive symptoms at T3 (β_T2-T3_ = −0.053, P < 0.001). Bootstrap results further confirmed the significant indirect effect of T1-CF on T3-depressive symptoms through T2-PA (β = −0.002, 95% CI=[−0.004, −0.001]), with the mediation effect accounting for 5.71%. However, the indirect effect of T1-CF on T3 depressive symptoms through T2-PA was not significant (β = −0.001, 95% CI=[−0.002, 0]).

**Conclusion:**

CF was negatively correlated with depressive symptoms. PA mediated the positive effect of CF on depressive symptoms in middle-aged and older adults, but failed to alleviate the negative effect of depression on CF. This study indicates that active participation in PA contributes to maintaining cognitive and mental health in middle-aged and older adults.

## Introduction

Cognitive function (CF) refers to the psychological functions of the human brain in recognizing and reflecting objective phenomena, including memory, thinking, sensation, language and understanding, among other abilities. It is a higher-level neural activity of the cerebral cortex [1]. As people grow older, their cognitive function undergoes dynamic changes, which may include normal cognitive ageing and the development of cognitive impairment. Cognitive impairment is a condition characterized by progressive and acquired neurocognitive decline, which is common among the elderly [2]. Generally, cognitive impairment encompasses two main stages: mild cognitive impairment (MCI) and dementia. Mild cognitive impairment usually precedes dementia and is characterized by a decline in cognitive function while daily functioning remains largely intact [3]. Mild cognitive impairment is a risk factor for dementia, with the risk increasing with the number of impaired cognitive domains and the severity of symptoms. It is conservatively estimated that the annual conversion rate from mild cognitive impairment to dementia is 5–10%. Currently, there are around 55 million people living with dementia worldwide, a figure which is expected to rise to 152 million by 2050 [4]. Dementia is associated with a reduced quality of life, disability and dependence on others in later life, as well as an increased risk of death. In light of the significant public health implications of dementia and the current absence of effective treatments, it is of critical importance to identify bio-psycho-social factors that can prevent or at least slow the progression of cognitive impairment in later life [5].

The global population is ageing, and this trend has accelerated since the beginning of the 21st century. According to the 2022 report World Population Prospects, the proportion of the global population aged 65 and over is expected to increase from 10% to 16% between 2022 and 2050 [6]. Against this backdrop, various age-related health issues have placed a significant burden on public health and healthcare systems, becoming a long-standing concern and urgent issue for the scientific community. As population ageing intensifies, depression among the elderly has become increasingly prevalent [7]. Depressive symptoms constitute a syndrome centered on depressive mood, primarily manifesting as low mood, anhedonia, physical discomfort, reduced appetite, sleep disturbances, and other psychological, physical and behavioral symptoms [8]. Depressive symptoms represent a sub-health state characterized by low cure rates, recurrent episodes and poor prognosis. If not managed promptly and effectively, they may progress to depression over time and, in some cases, lead to treatment-resistant depression [9]. The World Health Organization (WHO) predicts that depression will become the leading cause of global disease burden by 2030. The rising proportion of older adults, a high-risk group for depression, is a key factor in the increasing incidence of depression and disease burden [10].

A large body of evidence suggests that depressive symptoms are closely associated with cognitive impairment. On the one hand, cognitive impairment may contribute to the development of depressive symptoms. For example, a 15-year cohort study demonstrated a significant association between cognitive impairment at the start of the study and subsequent depressive symptoms in older adults [11]. Similarly, a prospective cohort study in the United States found that baseline cognitive deficits were associated with severe depressive symptoms that persisted at follow-up assessments. Conversely, depressive symptoms have been linked to cognitive decline and an increased risk of dementia [12]. Chen et al. conducted a 10-year follow-up study of 7,610 older adults with an average age of 65, finding that depressive symptoms were significantly associated with cognitive decline. The cumulative burden of depressive symptoms was independently and closely associated with poorer cognitive function, predicting accelerated cognitive decline in a dose-response pattern [13]. In fact, depressive symptoms in older adults exhibit high heterogeneity. According to the Diagnostic and Statistical Manual of Mental Disorders (DSM) and the International Classification of Diseases (ICD), these symptoms can be categorized as either somatic (e.g., changes in appetite, fatigue and sleep disturbances) or emotional (e.g., low mood and suicidal thoughts) [14]. Different symptom presentations may reflect different underlying mechanisms and may be linked to different aspects of cognitive impairment. Specifically, somatic depressive symptoms, such as fatigue and sleep disturbance, appear to be more closely associated with cognitive impairment than emotional symptoms, such as low mood or sadness [15]. To date, few longitudinal studies have examined the temporal relationship between specific types of depressive symptoms and CF, and the direction of the association between the two remains unclear.

In response to the aforementioned situation, the World Health Organization (WHO) states in its Guidelines on Physical Activity and Sedentary Behavior that physical activity can improve the functions and structures of the brain and nervous system, while the brain also plays a crucial role in regulating various aspects of physical activity [16]. Currently, numerous studies have identified a potential bidirectional association between CF and PA. The impact of CF on maintaining PA can be explained by experimental and theoretical research related to the effort minimization theory. PA can improve CF by promoting neurogenesis, angiogenesis and synaptic plasticity, as well as reducing pro-inflammatory processes and mitigating cellular damage caused by oxidative stress [17]. Some longitudinal studies have also demonstrated an association between PA and depression. Researchers have proposed several possible hypotheses. The protective hypothesis suggests that PA reduces depressive symptoms through biological mechanisms, such as increased endorphins, serotonin or endocannabinoids, or social mechanisms, such as social interaction and increased self-esteem [18]. In contrast, the inhibition hypothesis explains the inverse relationship, whereby depression negatively impacts physical activity through mechanisms such as anhedonia, low mood, and social withdrawal [19]. Overall, existing evidence suggests that PA may serve as a potential behavioral pathway linking CF and depressive symptoms over time [20]. However, no studies have yet explored whether PA mediates the relationship between CF and specific depressive symptom domains.

Based on this, the present study uses data from three English Longitudinal Study of Ageing (ELSA) surveys conducted between 2014 and 2018. Focusing on middle-aged and older adults aged 50 and above, the study employs the Cross-Lagged Panel Model (CLPM) for longitudinal analysis [21]. The aim is to explore the causal relationship between CF and depressive symptoms over time, and analyze the potential mediating role of PA in this bidirectional relationship. Understanding PA’s role in CF and depressive symptoms is crucial for developing and implementing preventive public health interventions targeting mental health disorders among middle-aged and older adults.

## Materials and methods

### Participants

**Fig 1:**
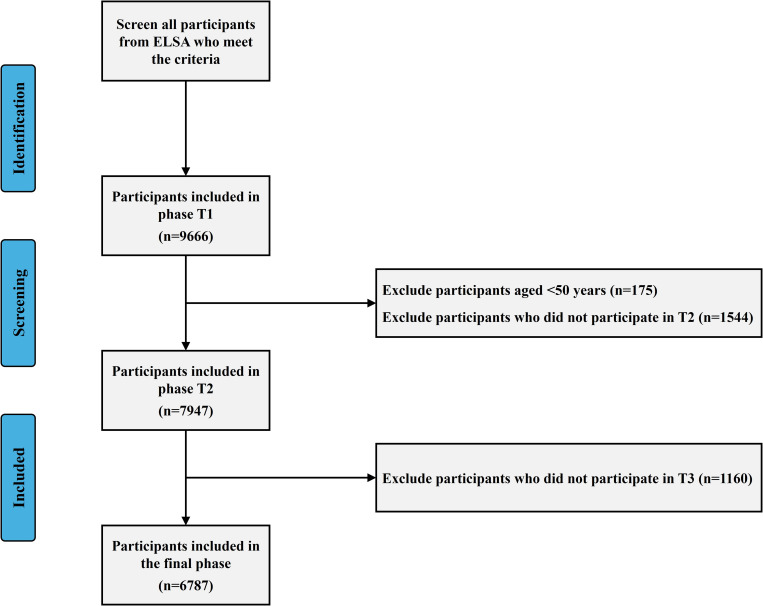
Research subject selection flowchart.

ELSA is a longitudinal survey study that aims to collect information on the health, social, and economic lives of people aged 50 and older in the UK [22]. The study began in 2002 with an initial sample of 12099 participants, who were contacted every two years for follow-up interviews. Data was collected using Computer-Assisted Personal Interviewing (CAPI) and questionnaires. ELSA was reviewed and approved by the National Research Ethics Committee, and all participants signed informed consent forms. Detailed information and data about ELSA can be obtained from the official website (https://beta.ukdataservice.ac.uk/datacatalogue/series/series?id=200011, Access data: GN 33368–5050-English Longitudinal Study of Ageing: Waves 0–11, 1998–2024) [22]. In this study, we used information collected in 2014–2015 (T1), 2016–2017 (T2), 2018–2019 (T3) because they contain the most recent available data sets on cognitive function, physical activity, and depressive symptoms. Among them, T1 is considered the baseline, and T2 and T3 are considered follow-ups. In ELSA, a total of 9666 study subjects participated in the T1 survey. This study excluded the following study subjects: (1) participants aged <50 years (=175), (2) participants who were lost to follow-up during the T2 and T3 periods (n = 2704). After screening, this study ultimately included 6787 participants who took part in all three surveys. The inclusion and exclusion process are shown in Fig 1.

### Procedures

This study measured two dimensions of cognitive function and depressive symptoms at three points and constructed a capital asset pricing model (CLPM) to examine the temporal relationship between cognitive function and emotional and physical depressive symptoms. Separate models were specified for affective depressive symptoms and somatic depressive symptoms. Subsequently, Wald χ2 tests, as part of the standard statistical output, were used to compare differences in cross-lagged paths within the models to identify the primary causal temporal relationships.

### Measurements

#### Cognitive function assessment.

Measuring cognitive functions include three aspects: working memory, cognitive flexibility, and executive function [23]. Working memory is assessed through a word recall test. Staff provide the subject with a list of 10 words and then ask the subject to immediately repeat as many words as possible within a time limit of 2 minutes (immediate memory). At the end of the cognitive unit, the subject is asked to repeat the words again, also within a time limit of 2 minutes. Correctly repeating a word earns one point. Memory scores are the sum of immediate memory and delayed memory scores, ranging from 0 to 20. Cognitive flexibility is assessed based on the participant’s responses to four date-related questions: day, month, year, and day of the week, with scores ranging from 0 to 4 points. Executive function is assessed through a verbal fluency task, which requires participants to list as many animal names as possible within one minute. The total number of animals mentioned constitutes the verbal fluency score.

Following the original scoring strategy used in ELSA-based cognitive research, the overall cognitive function score was calculated by summing the raw scores of the three domains. This approach preserves the original point-based interpretation of the cognitive tests and has been used in previous longitudinal aging cohort studies that combined memory, orientation, and executive function measures into a global cognitive function score [24]. We did not use a standardized or weighted composite score as the primary outcome because such an approach would transform the outcome into a sample-distribution-dependent relative measure and could overemphasize domains with restricted ranges or ceiling effects.

### Physical activity level

Self-reported PA is measured using three questions regarding the frequency of engaging in vigorous, moderate, and light PA (once a week or more, once a week, one to three times a month, or rarely). ELSA provides participants with examples of PA for each intensity category. Light physical activity (LPA) includes laundry and home repairs. Moderate physical activity (MPA) includes gardening, brisk walking, dancing, free gymnastics, or stretching exercises. Vigorous physical activity (VPA) includes running, jogging, swimming, cycling, aerobics, fitness exercises, tennis, etc. Based on the responses of the study participants, we further divided PA into four groups: sedentary group (SG): less than one session of LPA per week, no MPA or VPA. Low PA level group (LG): at least one session of LPA per week, one or less sessions of MPA per week, no VPA. Medium PA level group (LG): more than one session of MPA per week, one or less sessions of VPA per week. High PA level group (LG): more than one session of VPA per week.

### Depression symptoms evaluation

Depressive symptoms were assessed using the 8-item Center for Epidemiological Studies Depression Scale (CES-D), which has good reliability and validity [25]. Participants were asked whether they had experienced the following feelings in the past week: (a) low mood, (b) loneliness, (c) sadness, (d) enjoyment of life, (e) happiness, (f) lack of energy, (g) sleep disturbances, and (h) difficulty performing tasks. The six negative items were coded as: yes = 1, no = 0. The two positive items were coded in reverse. Based on symptom characteristics, we distinguished two dimensions from the CES-D items: affective depressive symptoms and somatic depressive symptoms. Affective depressive symptoms mainly reflect mood-related and emotional experiences, including low mood, loneliness, sadness, reduced enjoyment of life, and reduced happiness. Somatic depressive symptoms mainly reflect physical or functional manifestations of depression, including lack of energy, sleep disturbances, and difficulty performing tasks. In this study, affective depressive symptoms included items a, b, c, d, and e, while somatic depressive symptoms included items f, g, and h. The scoring ranges for affective depressive symptoms and somatic depressive symptoms were 0–5 points and 0–3 points, respectively. Generally, higher scores indicate more severe depressive symptoms.

### Covariance analysis

The covariates in this study include age, gender, race, marital status, employment status, educational attainment, smoking status, drinking status, BMI, and chronic diseases. Marital status: Participants were categorized as “single” or “married” based on their current marital status. “Single” includes “widowed”, “separated”, “divorced”, and “never married”; “married” includes “married” and “cohabiting”. Employment status: Participants were categorized as “employed” or “unemployed” based on their current employment status. Educational attainment: ELSA recorded individuals’ educational attainment through questionnaires and divided it into three levels: higher education, including National Vocational Qualification (NVQ) levels 4–5; secondary education, including NVQ levels 1–3; and lower education, referring to those without formal qualifications. Smoking status: Respondents were divided into two groups, “currently non-smokers” and “current smokers”, based on their answers to the question, “Do you currently smoke?” Drinking habits: Respondents’ drinking habits were categorized into three groups based on their answers to the question, “How often have you consumed alcoholic beverages in the past 12 months?” The categories were: rarely drinking alcohol (twice a year or less), moderate drinking (more than twice a year but less than three times a week), excessive drinking (three or more times per week). The latest BMI data was measured in 2012–2013, with height and weight obtained by professionals using tools. Age, marital status, education level, employment status, smoking, drinking, and chronic diseases were considered time-varying covariates and included in subsequent analyses.

### Cross-lagged panel model construction

CLPM is an analytical method that uses panel data from two or more longitudinal queues to explore the mutual relationships and directional influences between variables over time [26]. The path coefficients estimated by this model have clear time series relationships, which are consistent with the epidemiological principle of “cause before effect” in causal inference. In an ideal model, we assume that the respective regression and cross-lagged path coefficients are equal across time. The relationships between variables in CLPM can be described by the following equation:


$$\begin{array}{c} X_{t}=\beta_{x}X_{t-\text{1}}+b_{\text{1}}Y_{t-\text{1}} \end{array}$$

$$\begin{array}{c} Y_{t}=\beta_{y}Y_{t-\text{1}}+a_{\text{1}}X_{t-\text{1}} \end{array}$$

Among these, βx and βy are autoregressive coefficients that describe the stability of the variables themselves. a_1_ and b_1_ are cross-lagged coefficients that represent the effect of one variable on another variable in the lagged period after controlling for the predictive effect of the variable itself. The subscript t-1 indicates the time point of repeated measurement. To simplify the expression, the intercept terms and residual terms are not included in each equation.

The temporal relationship between the two variables X and Y is determined by comparing the absolute magnitudes of the cross-lagged path coefficients, which includes the following cases: If a_1_ = 0 and b_1_ = 0, there is no temporal relationship between X and Y. If a_1_ ≠ 0 and b_1_ = 0, the two variables have a unidirectional temporal relationship X → Y. If a_1_ = 0 and b_1_ ≠ 0, then the two variables have a unidirectional temporal relationship from Y to X. If a_1_ ≠ 0 and b_1_ ≠ 0, then the two variables have a bidirectional relationship of mutual regulation. At this point, if a_1_ > b_1_ and the difference between the two is significant, the main effect can be further determined as X → Y; otherwise, it is Y → X [27].

### Vertical intermediary model analysis

Mediation refers to the indirect influence of one or more variables M on the effect of an independent variable on a dependent variable. In this case, variable M is referred to as the mediating variable, and the role it plays is called the mediation effect. In practice, it is recommended to use longitudinal data to establish mediating relationships [28]. In longitudinal panel designs, the most commonly used mediation analysis model is the CLPM. It not only considers the temporal sequence required for testing causal inference, but also controls for the prior levels of mediating variables and dependent variables, providing strong evidence for revealing the causal direction and mediating mechanisms between variables. The relationship between variables in the longitudinal mediation analysis model can be described by the following equation:


$$\begin{array}{c} X_{t}=\beta_{x}X_{t-\text{1}}+b_{\text{2}}M_{t-\text{1}}+c_{\text{2}}Y_{t-\text{1}} \end{array}$$

$$\begin{array}{c} M_{t}=a_{\text{1}}X_{t-\text{1}}+\beta_{m}M_{t-\text{1}}+a_{\text{2}}Y_{t-\text{1}} \end{array}$$

$$\begin{array}{c} Y_{t}=c_{\text{1}}X_{t-\text{1}}+b_{\text{1}}M_{t-\text{1}}+\beta_{y}Y_{t-\text{1}} \end{array}$$

Similarly, the coefficients for the autoregressive and cross-lagged path effects are set to be time-invariant. The autoregressive effects between the same variables at different time points are represented by the coefficients β_x_, β_m_, and β_y_. The cross-lagged effects between different variables at different time points are represented by the coefficients a_1_, a_2_, b_1_, b_2_, c_1_, and c_2_. t-1 represents the time point, and the intercept and residual terms are not included in the equations. In this mediation model, indirect effects (longitudinal mediation effects) are represented by a_1_*b_1_ (corresponding to the path X_1_ → M_2_ → Y_3_) and a_2_*b_2_ (corresponding to the path Y_1_ → M_2_ → X_3_).

### Statistical analysis

Age, BMI, cognitive function, emotional depressive symptoms, and physical depressive symptoms are quantitative data but do not follow a normal distribution; they are described using the median (interquartile range). Gender, race, marital status, employment status, educational level, smoking status, drinking status, chronic diseases, and physical activity are categorical data; they are described using frequency and percentage. Correlation analysis was conducted on cognitive function, physical activity, emotional depressive symptoms, and physical depressive symptoms at T1, T2, and T3. If two variables simultaneously met the criteria for normal distribution, Pearson correlation analysis was used. If not, Spearman correlation analysis was used.

All models were evaluated using model fit statistics: Root Mean Squared Error of Approximation (RMSEA) ≤ 0.05, Comparative Fit Index (CFI), and Tucker-Lewis Index (TLI) ≥ 0.95, indicating good fit. RMSEA ≤ 0.08, CFI, and TLI > 0.90 are considered acceptable fits. To obtain the most parsimonious model, we compared the model fit of constrained models with that of unconstrained models, assessing differences in model fit based on changes in △RMSEA ≥ 0.015 and △CFI ≤ −0.01. Robust Maximum Likelihood Estimate (MLR) was used to estimate models with continuous variables. Mean and Weighted Least Squares with Mean and Variance Adjusted (WLSMV) were used to estimate models with categorical variables.

To investigate the impact of missing data, we imputed missing data five times using multiple imputation, repeated the main analysis on the five complete datasets generated, and summarized the results of the complete datasets according to Rubin’s rule to ultimately generate the estimated values of the summary model. All data analysis in this study was performed using SPSS_21.0 and Mplus_8.3. All tests were two-sided, and differences were considered statistically significant at P < 0.05.

## Results

### Basic characteristics of study subjects in the study

This study included a total of 6787 participants aged 50 years or older, of whom 3777 (55.7%) were women. The median age (interquartile range) of participants at baseline was 66 years. Table 1 presents the baseline characteristics of the participants. Cognitive function scores and emotional depression symptom scores remained relatively stable across the three surveys, with median scores of 37 and 0, respectively. In contrast, the median score for physical depression symptoms increased from 0 at T1 and T2 to 1 at T3. Over time, participants’ physical activity levels decreased slightly, with the percentage of sedentary individuals increasing from 5.3% at T1 to 8.7% at T3. See Table 1 for details.

**Table 1:** Basic characteristics of research subjects.

Parameters		T1	T2	T3
Age (median, interquartile range)		66(13)	68(13)	70(13)
BMI (kg/m², median, interquartile range)		27.1 (5.86)	27.7 (6.13)	26.9 (6.01)
Gender (n, %)	Male	3010 (44.3)		
	Female	3777 (55.7)		
Race (n, %)	White person	6545 (96.4)		
	Non-white	241 (3.6)		
Marital status (n, %)	Married	4992 (73.6)	4885 (72.0)	4771 (70.3)
	Unmarried	1795 (26.4)	1902 (28.0)	2016 (29.7)
Education level (n, %)	Low	1372 (20.8)	1364 (20.8)	1263 (19.0)
	Medium	3058 (46.3)	3076 (46.8)	2799 (42.1)
	High	2171 (32.9)	2128 (32.4)	2591 (38.9)
Employment situation (n, %)	Employed	2255 (33.9)	1855 (27.9)	1559 (23.3)
	Unemployed	4400 (66.1)	4793 (72.1)	5119 (76.7)
Smoking habit (n, %)	Smoking	698 (10.3)	631 (9.3)	573 (8.4)
	Non-smoking	6088 (89.7)	6130 (90.7)	6213 (91.6)
Chronic disease	0	2144 (31.6)	1972 (29.2)	1824 (27.0)
	1	2336 (34.5)	2327 (34.4)	2289 (33.8)
	≥2	2296 (33.9)	2465 (36.4)	2655 (39.2)
Cognitive function (median, interquartile range)		37 (12)	37 (12)	37 (13)
Physical activity level	Sedentary	358 (5.3)	439 (6.5)	588 (8.7)
	Low	1459 (21.5)	1566 (23.1)	1735 (25.6)
	Medium	3460 (51.0)	3366 (49.6)	3083 (45.4)
	High	1508 (22.2)	1415 (20.9)	1379 (20.3)
Depression symptom (median, interquartile range)	Emotional depression symptom	0 (0)	0 (1)	0 (1)
	Physical depression symptom	0 (1)	0 (1)	1 (1)

The results indicate that chronic disease burden was common among the study participants and showed a gradual increase over time. At T1, 2144 participants (31.6%) reported no chronic disease, 2336 (34.5%) reported one chronic disease, and 2296 (33.9%) reported two or more chronic diseases. At T2 and T3, the proportion of participants with two or more chronic diseases increased to 36.4% and 39.2%, respectively, while the proportion without chronic disease decreased from 31.6% at T1 to 27.0% at T3. These findings indicate that multimorbidity became more prevalent during follow-up, which is consistent with the ageing characteristics of the cohort. Chronic disease status was therefore included as a time-varying covariate in the subsequent analyses.

### Correlation analysis between different variables

**Fig 2:**
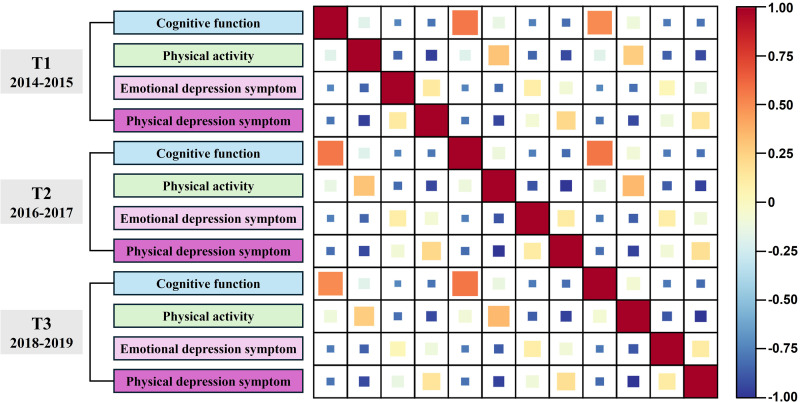
Correlation matrix of variables at different stages.

The results showed that there were significant correlations between CF, PA, emotional depression symptoms, and physical depression symptoms at all three time points (P < 0.001). Specifically, at the same time point and across time points, CF was significantly positively correlated with PA (r_s_=[0.247, 0.322]; P < 0.001), emotional depressive symptoms (r_s_=[−0.096, −0.131]; P < 0.001), and physical depressive symptoms (r_s_=[−0.132, −0.159]; P < 0.001); PA was significantly negatively correlated with emotional depressive symptoms (r_s_=[−0.150, −0.206]; P < 0.001) and physical depressive symptoms (r_s_=[−0.225, −0.266]; P < 0.001); affective depressive symptoms and somatic depressive symptoms were significantly positively correlated (r_s_=[0.281, 0.447]; P < 0.001). See Table 2 and Figure 2 for details.

**Table 2:** Correlation matrix between variables in each variable.

		T1				T2				T3			
		CF	PA	EDS	PDS	CF	PA	EDS	PDS	CF	PA	EDS	PDS
T1	CF	1**											
	PA	0.251*	1**										
	EDS	−0.102*	−0.17*	1**									
	PDS	−0.136*	−0.248*	0.447*	1**								
T2	CF	0.728**	0.247*	−0.104*	−0.132*	1**							
	PA	0.278*	0.563**	−0.157*	−0.232*	0.298*	1**						
	EDS	−0.115*	−0.171*	0.431*	0.321*	−0.118*	−0.206*	1**					
	PDS	−0.152*	−0.226*	0.316*	0.505**	−0.155*	−0.265*	0.44*	1**				
T3	CF	0.689**	0.252*	−0.096	−0.136*	0.73**	0.286*	−0.125*	−0.149*	1**			
	PA	0.303*	0.539**	−0.15*	−0.226*	0.312*	0.59**	−0.185*	−0.242*	0.322*	1**		
	EDS	−0.123*	−0.176*	0.388*	0.3*	−0.123*	−0.189*	0.433*	0.31*	−0.131*	−0.199*	1**	
	PDS	−0.139*	−0.225*	0.281*	0.465*	−0.144*	−0.241*	0.307*	0.483*	−0.159*	−0.266*	0.439*	1**

Note: * = p < 0.05, ** = p < 0.01, CF: Cognitive function, PA: Physical activity, EDS: Emotional depression symptom, PDS: Physical depression symptom.

### Fitting of longitudinal association models between cognitive function, physical activity, and depressive symptoms

This study used CLPM to investigate the causal temporal relationship between CF and depressive symptoms in older adults, as well as the mediating role of PA in this association. To simplify the model, unconstrained models (Model 1a/Model 2a) and constrained models (Model 1b/Model 2b) were compared based on △RMSEA ≥ 0.015 and △CFI ≤ −0.01. In addition, the basic characteristics reported earlier were incorporated into the CLPM as covariates to account for their potential confounding effects, and the corresponding adjustments have been specified in both the methodological description and the results section.

The results showed that in CLPM, Model 1b had better model fit than Model 1a (△RMSEA = −0.002, △CFI = 0.001). Therefore, Model 1b was selected as the final model, which had good model fit (RMSEA = 0.043, CFI = 0.916, TLI = 0.855). In the mediation model, there was no significant difference in model fit between Model 2a and Model 2b (△RMSEA = −0.001, △CFI = −0.003). Based on the principle of model parsimony, this study selected the constrained model, i.e., Model 2b, as the final model, with model fit indices of: RMSEA = 0.035, CFI = 0.935, TLI = 0.886. These results indicate good model fit. Detailed results are presented in Table 3.

**Table 3:** Correlation matrix between variables in each variable.

Model parameter		Model fit			Model comparison	
		RMSEA	CFI	TLI	RMSEA	CFI
Cross-lagged panel model	Model-1a	0.045	0.915	0.846		
	Model-1b	0.043	0.916	0.855	−0.002	0.001
Longitudinal mediation analysis model	Model-2a	0.036	0.938	0.884		
	Model-2b	0.035	0.935	0.886	−0.001	−0.003

Note: RMSEA = Root Mean Square Error of Approximation, CFI = Comparative Fit Index, TLI = Tucker-Lewis Index, Model-1a/ Model-2a = Unrestricted Model, Model-1b/Model-2b = Constrained autoregressive path model and Cross-lagged panel model.

### Analysis of the causal temporal relationship between cognitive function and depressive symptoms

**Fig 3:**
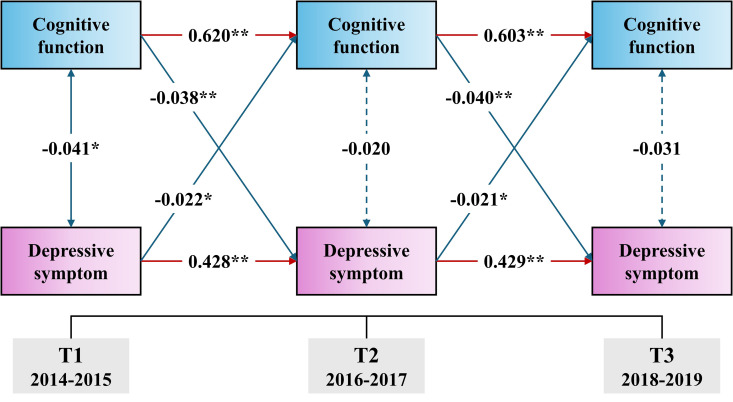
Results of cross-lagged tests for cognitive function and depressive symptoms. Note: * = P < 0.05, * = P < 0.01. Solid lines indicate statistical significance, while dashed lines indicate no statistical significance.

The CLPM results for CF and depressive symptoms showed that the autoregressive effects of CF and depressive symptoms were significant (P < 0.001) and stable across time. In other words, past CF and depressive symptoms predicted future CF and depressive symptoms, respectively. There is a significant negative lag effect from CF to depressive symptoms (β_T1-T2_ = −0.038, P < 0.001; β_T2-T3_ = −0.040, P < 0.001). This also indicates that poorer CF predicts more severe depressive symptoms in the future. The reverse cross-lagged effect from depressive symptoms to CF also showed a significant negative correlation (β_T1-T2_ = −0.022, P = 0.005; β_T2-T3_ = −0.021, P = 0.005). This indicates that more severe depressive symptoms predict poorer CF levels in the future. Statistically significant cross-lagged paths indicate a bidirectional longitudinal association between CF and depressive symptoms. Wald tests revealed that the path from CF to depressive symptoms was significantly stronger than the path from depressive symptoms to CF (Wald χ² = 7.546, P = 0.006). That is, in the bidirectional relationship between CF and depressive symptoms, CF is the dominant factor. Detailed results are shown in Fig 3.

### Longitudinal mediated effect of physical activity on the relationship between cognitive function and depressive symptoms

**Fig 4:**
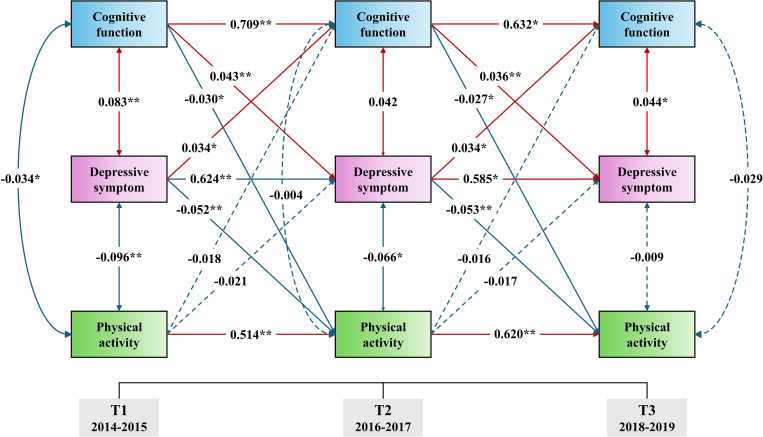
Results of cross-lagged tests for cognitive function and depressive symptoms. Note: * = P < 0.05, * = P < 0.01. Solid lines indicate statistical significance, while dashed lines indicate no statistical significance.

This study constructed a cross-lagged mediation model to investigate whether PA mediates the relationship between CF and depressive symptoms. The results showed that poorer CF at T1 predicted lower levels of PA at T2 (β_T1-T2_ = 0.043, P < 0.001), and lower levels of PA at T2 predicted more severe depressive symptoms at T3 (β_T2-T3_ = −0.053, P < 0.001). These path coefficients supported the temporal ordering of the hypothesized CF → PA→depressive symptoms pathway, while evidence for mediation was determined based on the bootstrap indirect effect and its 95% confidence interval reported below. To further test the longitudinal mediation, this study used a bias-corrected Bootstrap method with 5000 resamples to determine the indirect effect of T1-CF on T3-depressive symptoms through T2-PA. The results showed that the indirect effect between variables was significant (β = −0.002, 95% CI=[−0.004, −0.001]). That is, PA mediated the effect of CF on depressive symptoms, with the mediating effect accounting for 5.71%. However, this study did not find evidence of reverse mediation. Specifically, the severity of T1-depressive symptoms did not predict T2-PA levels (β_T1-T2_ = −0.021, P = 0.075), although lower T2 PA levels were associated with poorer T3 CF (β_T2-T3_ = 0.034, P < 0.05). Additionally, the indirect effect of T1-depressive symptoms on T3-cognitive function through T2-PA was not significant (β = 0.001, 95% CI=[−0.002, 0]). Detailed results are presented in Table 4 and Fig 4.

**Table 4:** Statistical results of longitudinal mediation analysis.

Path parameters	β	95%CI		Mediation effect proportion
		Lower limit	Upper limit	
T1-CF → T2-PA → T3-Depression	−0.002	−0.004	−0.001	5.71
T1-Depression→T2-PA → T3-CF	−0.001	−0.002	0	/

### Sensitivity analysis results of longitudinal mediated and cross-lagged models

In addition, this study used sensitivity analysis and multiple imputations to fill in missing values. Specifically, we conducted sensitivity analyses by comparing results obtained from complete-case data with those from data after multiple imputations, and by estimating models with and without covariates to examine the stability of the core temporal associations. The results showed that PA mediated the bidirectional association between CF and depressive symptoms across these different analytic specifications, indicating that the findings are robust to alternative model settings and missing data handling. Specific results showed that CF significantly predicted depressive symptoms at T2 (β = −0.052, SE = 0.008, P < 0.001), and T2-CF also negatively predicted T3-Depression (β = −0.054, SE = 0.009, P < 0.001). The reverse path was also statistically significant, with T1-Depression significantly negatively predicting T2-CF (β = −0.026, SE = 0.006, P < 0.001), and the effect of T2-Depression on T3-CF was β = −0.025 (SE = 0.006, P < 0.001). These results support a dynamic interactive relationship between CF and depressive symptoms in middle-aged and older adults. The model fit was good (RMSEA = 0.045, CFI = 0.924, TLI = 0.869), indicating that the cross-lagged path settings were reasonable.

In the longitudinal mediation model further introducing PA as a mediating variable, the results showed that PA partially mediated the relationship between CF and depressive symptoms. Specifically, PA mediated the time-ordered pathway from T1 cognitive function to T3 depressive symptoms through T2 physical activity. In the longitudinal mediation model further introducing PA as a mediating variable, the sensitivity analysis was interpreted based on the two prespecified time-ordered indirect pathways rather than on the statistical significance of individual cross-lagged paths alone. The indirect pathway from T1-CF to T3 depressive symptoms through T2-PA remained significant (β = −0.002, 95% CI=[−0.004, −0.001]), indicating that PA mediated the longitudinal association from earlier CF to later depressive symptoms.

In contrast, the reverse indirect pathway from T1 depressive symptoms to T3-CF through T2-PA was not significant (β = −0.001, 95% CI=[−0.002, 0]), indicating that the sensitivity analysis did not support a reverse mediation pathway. The model fit indices were good (RMSEA = 0.035, CFI = 0.942, TLI = 0.898), suggesting that the sensitivity analysis was consistent with the main mediation findings. Detailed results are presented in Table 5.

**Table 5:** Standardized path coefficients and model fit indices for longitudinal mediated and cross-lagged models.

Model parameter	Path parameters	β	SE	P
Cross-lagged panel model	T1-CF → T2-Depression	−0.052	0.008	<0.001
	T2-CF → T3-Depression	−0.054	0.009	<0.001
	T1-Depression→T2-CF	−0.026	0.006	<0.001
	T2-Depression→T3-CF	−0.025	0.006	<0.001
Longitudinal mediation analysis model	T1-CF → T2-PA	0.083	0.012	<0.001
	T2-CF → T3-PA	0.068	0.008	<0.001
	T1-PA → T2-CF	0.06	0.01	<0.001
	T2-PA → T3-CF	0.053	0.009	<0.001
	T1-CF → T2-Depression	−0.045	0.01	<0.001
	T2-CF → T3-Depression	−0.04	0.008	<0.001
	T1-Depression→T2-CF	−0.023	0.008	0.007
	T2-Depression→T3-CF	−0.017	0.006	0.007
	T1-Depression→T2-PA	−0.042	0.01	<0.001
	T2-Depression→T3-PA	−0.033	0.008	<0.001
	T1-PA → T2-Depression	−0.055	0.011	<0.001
	T2-PA → T3-Depression	−0.056	0.01	<0.001

Note: The model fitting for the cross-lagged panel model is: RMSEA = 0.045, CFI = 0.924, TLI = 0.869; the model fit for the longitudinal mediation model is: RMSEA = 0.035, CFI = 0.942, TLI = 0.898.

## Discussion

This study was based on longitudinal data from ELSA from 2014 to 2018, involving a total of 6,787 participants aged 50 and over. The study explored the relationship between CF and depressive symptoms in middle-aged and elderly people, as well as the role of PA in this relationship. Overall, there was a negative correlation between CF and depressive symptoms in both directions, with CF being the dominant factor. Mediational analysis indicates that CF can predict depressive symptoms via PA, but this effect is less stable over time in longitudinal studies. Conversely, depressive symptoms directly and stably predict CF in a negative direction. Therefore, this study revealed the direct and indirect effects of PA on CF and depressive symptoms, which are significant for formulating and implementing preventive public health interventions for mental health disorders among older adults in the community.

Both cross-sectional and longitudinal studies have demonstrated the existence of specific relationships between various dimensions of depressive symptoms and CF. Divers et al. conducted a cross-sectional study of 9154 patients aged 50 and over with mild cognitive impairment, finding that physical symptoms and anhedonia were associated with cognitive decline [29]. Baune et al. conducted a cross-sectional study of 365 older adults aged 65–83 and found that increased depressive mood and somatic symptoms were significantly associated with lower attention and motor function scores, whereas positive mood and interpersonal difficulties were not significantly associated with any cognitive domain [30]. Existing longitudinal studies typically focus on the unidirectional association between CF and depressive symptoms. For instance, a seven-year longitudinal study in the Netherlands involving 736 middle-aged and older adults with a history of vascular disease employed generalized estimating equation models to analyze the impact of CF on depressive symptoms. The results showed that poorer cognitive performance in the domains of memory, executive function and information processing speed was associated with higher levels of motivational and emotional symptoms [31]. A prospective study of 298 older African American adults with an average age of 74 found that anhedonia and negative emotions were associated with subsequent cognitive decline [32]. Relatively few studies have investigated the bidirectional association between CF and depressive symptoms in middle-aged and older adults to date. Teles et al. conducted a longitudinal study of 2057 older adults aged 60–99 years. After adjusting for gender and education, they found a unidirectional association from depressive symptoms to subsequent memory changes, i.e., higher somatic symptoms and depressive mood predicted subsequent memory decline, while higher positive mood predicted better subsequent memory performance. However, no opposite association was found [33].

Previous studies have shown that the association among CF, PA, and depressive symptoms in middle-aged and older adults may have a physiological basis. However, the mediation findings in the present study should be interpreted within the two specific time-ordered indirect pathways tested in the CLPM. Specifically, the pathway from T1-CF to T3 depressive symptoms through T2-PA was significant, indicating that poorer baseline CF predicted lower subsequent PA, which in turn predicted more severe later depressive symptoms. In contrast, the reverse mediated pathway from T1 depressive symptoms to T3-CF through T2-PA was not significant, suggesting that PA did not significantly transmit the effect of earlier depressive symptoms on later CF. Therefore, the mediating role of PA was mainly observed in the CF → PA→depressive symptoms pathway rather than in the reverse depressive symptoms→PA → CF pathway. For instance, the serotonin-norepinephrine system may be a common neural pathway underlying emotional and physical symptoms in patients with depressive disorders [34,35], and this system may be pivotal in the mechanisms of CF and neuronal degeneration [36]. Furthermore, the longitudinal association between CF and depressive symptoms indicates that promoting positive CF is a crucial intervention strategy for enhancing mental health in middle-aged and older adults [37]. Therefore, given the inhibitory effect of PA on depression and its regulatory role in the intracranial environment and small molecules, this study further emphasizes the importance of prioritizing and enhancing CF at the primary prevention level. This can be achieved through cognitive training, organizing cognitive stimulation activities and encouraging lifelong learning [38].

It is worth noting that this study also revealed that PA mediates the association between CF and depressive symptoms. A better understanding of these factors could significantly improve the management and prevention of mental health conditions. Indeed, there is a strong link between CF and PA, as well as between PA and depressive symptoms [39]. Firstly, this study found that poorer CF predicts lower PA levels, and conversely, lower PA levels predict poorer CF, thereby supporting and expanding upon the evidence from unidirectional association studies. For instance, a meta-analysis of 15 prospective studies revealed that individuals engaging in PA at baseline experienced a notably reduced risk of cognitive decline during the follow-up period [40]. Cheval et al. conducted a 13-year longitudinal study using a linear mixed-effects model with 104590 participants from the European Health, Ageing and Retirement Survey. They found that CF may help older adults maintain regular PA [41]. In recent years, bidirectional longitudinal studies have been conducted to explore the mutual influence between the two. However, the results of these studies are inconsistent. Some studies have found that PA levels can predict subsequent cognitive ability, but baseline cognitive ability cannot predict subsequent PA levels [42]. However, other studies have yielded opposite results: CF can predict subsequent PA levels, while PA status is not significantly associated with subsequent cognitive levels [43]. Furthermore, Ren conducted a systematic review incorporating 12 studies to clarify the bidirectional relationship between cognitive performance and PA. They found that long-term aerobic exercise can effectively reduce the incidence of severe depression in adults and improve cognitive function, as well as subdomains of memory and executive function [44]. These conflicting results may be due to methodological differences between studies, such as variations in participants’ age ranges and clinical characteristics.

Additionally, this study found that more severe depressive symptoms can predict lower levels of PA in the future. Some studies support the idea that participating in PA can improve different symptom dimensions of depression. A 12-year longitudinal study of 2660 Taiwanese adults aged 60 and over found that a decrease in PA between two surveys was associated with an increase in physical symptoms, higher CES-D scores and lower positive emotion scores at the follow-up survey. Conversely, an increase in PA was associated with higher positive emotion scores and lower CES-D scores at the follow-up survey [45]. Meanwhile, a prospective study of 212 veterans with chronic obstructive pulmonary disease (COPD), with an average age of 69, found that participants had severe depression and physical symptoms at baseline. Three months later, daily step counts and PA levels had decreased further, and some patients had reported additional somatization of depression or pain [46]. Although the underlying biological mechanisms are unclear, the lateral hypothalamic orexin pathway is a potential candidate. The orexin/hypothalamic secretin pathway is primarily involved in regulating sleep and wakefulness and feeding behavior, but it also receives somatosensory afferent input. Furthermore, orexin plays a pivotal role in the regulation of spontaneous PA [47]. This biological mechanism may partially explain the association between depressive symptoms and PA in this study. However, since previous studies have generally used total depressive symptom scores, differences in the various dimensions of depressive symptoms are often overlooked. This blanket approach may obscure the complexity and diversity of the different depressive symptom dimension [48]. This study distinguishes between emotional and somatic depressive symptoms, highlighting the necessity of considering the specific effects of depressive symptoms in future research. This distinction enables a more precise understanding of how depressive symptoms influence CF and other health outcomes, providing a scientific basis for developing more personalized and effective intervention measures.

Recent research from other countries has provided valuable context for our findings on PA, CF, and depressive symptoms in aging populations. For example, a longitudinal cohort study in the UK using ELSA data demonstrated that higher levels of physical activity were significantly associated with better global cognitive function and specific cognitive domains, and that depressive symptoms partially mediated this association, reinforcing the interrelated nature of PA, mood, and cognition in later life [49]. This aligns with our observation of temporal associations between PA, CF, and depressive symptoms in a community-based cohort. Additionally, cross-national analyses across multiple countries have reported that physical inactivity is prevalent among older adults and that higher PA levels are linked with lower depressive symptoms in high-income settings, though associations can vary by socio‑economic context [50]. These international findings echo our results and suggest that the relationships among PA, mood, and cognitive health are broadly observed across diverse aging populations. By situating our results within this broader literature, our study contributes to the growing evidence that promoting PA may support emotional and cognitive well‑being in older adults globally.

In summary, this study has three key advantages. Firstly, it is based on a large, nationally representative chore, providing solid data and strong statistical power. Secondly, it distinguishes specific dimensions of depressive symptoms, elucidating potential associations between CF, PA, and these dimensions. Thirdly, it adjusts for a series of covariates, controlling for confounding factors and enhancing the reliability and validity of the results. Based on the above results and discussions, the study indicates a bidirectional negative association between CF and depressive symptoms in middle-aged and older adults. CF was negatively correlated with depressive symptoms. PA temporally mediated the effect of CF on depressive symptoms, but did not significantly influence the reverse pathway from depressive symptoms to CF. These findings suggest that engaging in PA may help maintain cognitive and mental health in middle-aged and older adults, and that increasing PA levels and duration could further support this temporal relationship.

### Limitations and suggestions for future research

Although this study systematically examined the mediating effect of PA on the bidirectional association between CF and depressive symptoms in middle-aged and older adults, it still has certain limitations. Firstly, using self-reported data rather than objective measurement methods to assess cognitive function, physical activity, and depressive symptoms may introduce recall and social desirability biases.

Secondly, due to the limitations of the ELSA database, the analysis could not include or adjust for potential confounding factors such as life stress events, family history of mental disorders, or genetic characteristics, thereby potentially leaving residual confounding factors. Thirdly, the cross-lagged panel model cannot distinguish between differences within and between individuals. While derived models such as the random intercept cross-lagged panel model (RI-CLPM) could address this issue, we did not adopt this approach in the current study because our primary objective was to investigate the temporal associations and mediating role of PA using the available three-wave longitudinal data. Applying RI-CLPM with only three time points may result in unstable parameter estimates and reduce the interpretability of the model. Nevertheless, we acknowledge this as a limitation and suggest that future studies consider RI-CLPM or other appropriate models to disentangle within- and between-individual effects for more robust inference.

Finally, this study explored one possible mechanism underlying the relationship between cognitive function and depressive symptoms and found that physical activity had a mediating effect. While this effect was statistically significant, it accounted for only a small proportion of the overall effect, suggesting limited explanatory power. Further research is therefore needed to assess other potential mediating factors, in order to fully understand the complex mechanisms underlying the relationship between cognitive function and the various dimensions of depression.

## Conclusion

1. CF is negatively associated with depressive symptoms in middle-aged and older adults, with CF being the dominant factor.
2. CF temporally predicts depressive symptoms via PA, and the reverse pathway shows a temporal association as well.
3. CF appears to play a dominant role in the association between cognition and depression, highlighting PA as a potential underlying mechanism, particularly in middle-aged and older adults with chronic diseases and prolonged depressive symptoms.

## References

[1] Zeng Y, Feng Q, Hesketh T, Christensen K, Vaupel JW. Survival, disabilities in activities of daily living, and physical and cognitive functioning among the oldest-old in China: a cohort study. Lancet. 2017;389(10079):1619–29. doi: 10.1016/S0140-6736(17)30548-2 28285816 PMC5406246

[2] Porsteinsson AP, Drye LT, Pollock BG, Devanand DP, Frangakis C, Ismail Z, et al. Effect of citalopram on agitation in Alzheimer disease: the CitAD randomized clinical trial. JAMA. 2014;311(7):682–91. doi: 10.1001/jama.2014.93 24549548 PMC4086818

[3] Kingston A, Wohland P, Wittenberg R, Robinson L, Brayne C, Matthews FE, et al. Is late-life dependency increasing or not? A comparison of the Cognitive Function and Ageing Studies (CFAS). Lancet. 2017;390(10103):1676–84. doi: 10.1016/S0140-6736(17)31575-1 28821408 PMC5640505

[4] Anderer S. Lack of personal growth, purpose linked with mild cognitive impairment. JAMA. 2024;332(13):1044. doi: 10.1001/jama.2024.17611 39240558

[5] Rookes T, Frost R, Barrado-Martin Y, Marston L, Cooper C, Gardner B, et al. Type of goals set and progress towards these goals, as part of a behaviour change intervention, in people with mild cognitive impairment: a secondary analysis. Lancet. 2023;402 Suppl 1:S80. doi: 10.1016/S0140-6736(23)02112-8 37997126

[6] Asamani JA, Bediakon KSB, Boniol M, Munga’tu JK, Akugri FA, Muvango LL, et al. Projected health workforce requirements and shortage for addressing the disease burden in the WHO Africa Region, 2022-2030: a needs-based modelling study. BMJ Global Health. 2024;7(Suppl 1). doi: 10.1136/bmjgh-2024-015972 39438055 PMC11789529

[7] Global Nutrition Target Collaborators. Global, regional, and national progress towards the 2030 global nutrition targets and forecasts to 2050: a systematic analysis for the Global Burden of Disease Study 2021. Lancet. 2025;404(10471):2543–83. doi: 10.1016/S0140-6736(24)01821-X 39667386 PMC11703702

[8] Anderer S. US depression rates rise, with variation across sex. JAMA. 2025;333(23):2043–4. doi: 10.1001/jama.2025.6449 40377954

[9] Collins N. Groundbreaking studies expand genetic associations with depression and bipolar disorder. JAMA. 2025;333(17):1476–7. doi: 10.1001/jama.2025.2934 40184050

[10] Mokdad AH, Ballestros K, Echko M, Glenn S, Olsen HE, Mullany E. The State of US health, 1990-2016: burden of diseases, injuries, and risk factors among US States. JAMA. 2018;319(14):1444–72. doi: 10.1001/jama.2018.0158 29634829 PMC5933332

[11] Nartey Y, Chalitsios CV, Khan N, Simpson G, Dambha-Miller H, Farmer A. Factors associated with multimorbidity in England: an analysis of the English longitudinal study of ageing. Lancet. 2023;402 Suppl 1:S73. doi: 10.1016/S0140-6736(23)02126-8 37997118

[12] Barnett ML, Waken RJ, Zheng J, Orav EJ, Epstein AM, Grabowski DC, et al. Changes in health and quality of life in US skilled nursing facilities by COVID-19 exposure status in 2020. JAMA. 2022;328(10):941–50. doi: 10.1001/jama.2022.15071 36036916 PMC9425288

[13] Chen S, Kuper H. Tracing the temporal trends of modifiable risk factors in dementia: insights from the English Longitudinal Study of Ageing (2004-2019). Lancet. 2023;402 Suppl 1:S34. doi: 10.1016/S0140-6736(23)02078-0 37997075

[14] Huang W-L, Chiu Y-T, Löwe B, Wu C-S, Liao S-C. Psychopathologies and quality of life in mental and functional disorders associated with persistent somatic symptoms. J Affect Disord. 2025;387:119521. doi: 10.1016/j.jad.2025.119521 40441622

[15] O’Connor M, Vang ML, Bryant RA, Buur C, Komischke-Konnerup KB, Frostholm L, et al. Development and validation of the Aarhus Structured Clinical Interview for Prolonged Grief Disorder in ICD-11 and DSM-5-TR (A-PGDi). Eur J Psychotraumatol. 2025;16(1):2511373. doi: 10.1080/20008066.2025.2511373 40534423 PMC12180314

[16] Hua M, Hua Y, Peng Y, Zhu J. Associations between adherence to 24-hour movement guidelines with depression, anxiety, and loneliness among Chinese adolescents. J Affect Disord. 2025;385:119369. doi: 10.1016/j.jad.2025.05.029 40339715

[17] Tari AR, Walker TL, Huuha AM, Sando SB, Wisloff U. Neuroprotective mechanisms of exercise and the importance of fitness for healthy brain ageing. Lancet. 2025;405(10484):1093–118. doi: 10.1016/S0140-6736(25)00184-9 40157803

[18] Slomski A. Even low amounts of physical activity reduce depression risk. JAMA. 2022;327(21):2066. doi: 10.1001/jama.2022.8997 35670798

[19] Köhler-Forsberg O, Cusin C, Nierenberg AA. Evolving issues in the treatment of depression. JAMA. 2019;321(24):2401–2. doi: 10.1001/jama.2019.4990 31125042

[20] Csajbók Z, Sieber S, Cullati S, Cermakova P, Cheval B. Physical activity partly mediates the association between cognitive function and depressive symptoms. Transl Psychiatry. 2022;12(1):414. doi: 10.1038/s41398-022-02191-7 36167692 PMC9515096

[21] Vedder A, O’Connor M, Boelen PA. Emotional vs. social loneliness and prolonged grief: a random-intercept cross-lagged panel model. Eur J Psychotraumatol. 2025;16(1):2488101. doi: 10.1080/20008066.2025.2488101 40260969 PMC12016272

[22] Alattas A, Shuweihdi F, Best K, Nikolova S, West R. Bidirectional association between frailty and quality of life within English longitudinal study of aging. Quality of Life Research: An International J Quality of Life Aspects of Treatment, Care and Rehabilitation. 2025;34(1):261–71. doi: 10.1007/s11136-024-03809-7 39400690 PMC11802669

[23] Lam NH, Mukherjee A, Wimmer RD, Nassar MR, Chen ZS, Halassa MM. Prefrontal transthalamic uncertainty processing drives flexible switching. Nature. 2025;637(8044):127–36. doi: 10.1038/s41586-024-08180-8 39537928 PMC11841214

[24] Zheng G, Zhou B, Fang Z, Chen X, Liu M, He F, et al. Long-term visit-to-visit blood pressure variability and cognitive decline among patients with hypertension: a pooled analysis of 3 national prospective cohorts. J Am Heart Assoc. 2024;13(13):e035504. doi: 10.1161/JAHA.124.035504 38934858 PMC11255695

[25] Chen P, Chen G, Tang G, Yang Z, Ma W, Chen C, et al. Effects of light therapy on amygdala connectivity and serotoninergic system in young adults with subthreshold depression. Dialogues Clin Neurosci. 2025;27(1):184–200. doi: 10.1080/19585969.2025.2503367 40451207 PMC12128131

[26] Wu TC-H, Lloyd A, Viding E, Fearon P. Examining longitudinal associations between interpersonal outcomes and general psychopathology factors across preadolescence using random intercept cross-lagged panel model. J Child Psychol Psychiatry. 2025;66(7):932–45. doi: 10.1111/jcpp.14105 39731442 PMC12198929

[27] Liu N, Wang Y, Li Z. Internet use, physical activity and depressive symptoms in older adults: A cross-lagged panel analysis. J Affect Disord. 2024;350:937–45. doi: 10.1016/j.jad.2024.01.201 38278330

[28] The moderator–mediator variable distinction in social psychological research: Conceptual, strategic, and statistical considerations. 1986. 10.1037//0022-3514.51.6.1173 3806354

[29] Divers R, Robinson A, Miller L, Davis K, Reed C, Calamia M. Examining heterogeneity in depression symptoms and associations with cognition and everyday function in MCI. Journal of clinical and experimental neuropsychology. 2022;44(3):185–94. doi: 10.1080/13803395.2022.2102154 35862574 PMC9665159

[30] Baune BT, Suslow T, Arolt V, Berger K. The relationship between psychological dimensions of depressive symptoms and cognitive functioning in the elderly - the MEMO-study. J Psychiatr Res. 2007;41(3–4):247–54. doi: 10.1016/j.jpsychires.2006.06.004 16887147

[31] Caspersen CJ, Powell KE, Christenson GM. Physical activity, exercise, and physical fitness: definitions and distinctions for health-related research. Public Health Rep. 1985;100(2):126–31. 3920711 PMC1424733

[32] Hamer M, Chida Y. Physical activity and risk of neurodegenerative disease: a systematic review of prospective evidence. Psychol Med. 2009;39(1):3–11. doi: 10.1017/S0033291708003681 18570697

[33] The CES-D Scale: A self-report depression scale for research in the general population. US: Sage Publications; 1977.

[34] Harris E. Weaker link than expected between exercise and less cognitive decline. JAMA. 2024;331(9):725. doi: 10.1001/jama.2024.0354 38353976

[35] Harris E. Meta-analysis: exercise as effective as therapy for treating depression. JAMA. 2024;331(11):908. doi: 10.1001/jama.2024.1121 38416479

[36] Blough J, Loprinzi PD. Experimentally investigating the joint effects of physical activity and sedentary behavior on depression and anxiety: a randomized controlled trial. J Affect Disord. 2018;239:258–68. doi: 10.1016/j.jad.2018.07.019 30029153

[37] Cooper-Patrick L, Ford DE, Mead LA, Chang PP, Klag MJ. Exercise and depression in midlife: a prospective study. Am J Public Health. 1997;87(4):670–3. doi: 10.2105/ajph.87.4.670 9146452 PMC1380853

[38] Baumeister SE, Leitzmann MF, Bahls M, Dörr M, Schmid D, Schomerus G, et al. Associations of leisure-time and occupational physical activity and cardiorespiratory fitness with incident and recurrent major depressive disorder, depressive symptoms, and incident anxiety in a general population. J Clin Psychiatry. 2017;78(1):e41–7. doi: 10.4088/JCP.15m10474 28129498

[39] Zhao Y-L, Sun S-Y, Qin H-C, Zhu Y-L, Luo Z-W, Qian Y, et al. Research progress on the mechanism of exercise against depression. World J Psychiatry. 2024;14(11):1611–7. doi: 10.5498/wjp.v14.i11.1611 39564183 PMC11572674

[40] Northey JM, Cherbuin N, Pumpa KL, Smee DJ, Rattray B. Exercise interventions for cognitive function in adults older than 50: a systematic review with meta-analysis. Br J Sports Med. 2018;52(3):154–60. doi: 10.1136/bjsports-2016-096587 28438770

[41] Sanders LMJ, Hortobágyi T, la Bastide-van Gemert S, van der Zee EA, van Heuvelen MJG. Dose-response relationship between exercise and cognitive function in older adults with and without cognitive impairment: a systematic review and meta-analysis. PLoS One. 2019;14(1):e0210036. doi: 10.1371/journal.pone.0210036 30629631 PMC6328108

[42] Afanador-Restrepo DF, Casanova-Correa A, Martín-Ojeda RI, Aibar-Almazán A, González-Martín AM, Hita-Contreras F, et al. Dose-response relationship of high-intensity training on global cognition in older adults with mild cognitive impairment or dementia: a systematic review with meta-analysis - the ACHIEVE-Study. Eur Rev Aging Phys Act. 2024;21(1):23. doi: 10.1186/s11556-024-00358-3 39285266 PMC11407012

[43] Hoffmann CM, Petrov ME, Lee RE. Aerobic physical activity to improve memory and executive function in sedentary adults without cognitive impairment: a systematic review and meta-analysis. Prev Med Rep. 2021;23:101496. doi: 10.1016/j.pmedr.2021.101496 34377632 PMC8327129

[44] Ren F-F, Hillman CH, Wang W-G, Li R-H, Zhou W-S, Liang W-M, et al. Effects of aerobic exercise on cognitive function in adults with major depressive disorder: a systematic review and meta-analysis. Int J Clin Health Psychol. 2024;24(2):100447. doi: 10.1016/j.ijchp.2024.100447 38371396 PMC10869919

[45] Groot C, Hooghiemstra AM, Raijmakers PGHM, van Berckel BNM, Scheltens P, Scherder EJA, et al. The effect of physical activity on cognitive function in patients with dementia: a meta-analysis of randomized control trials. Ageing Res Rev. 2016;25:13–23. doi: 10.1016/j.arr.2015.11.005 26607411

[46] Song D, Yu DSF, Li PWC, Lei Y. The effectiveness of physical exercise on cognitive and psychological outcomes in individuals with mild cognitive impairment: a systematic review and meta-analysis. Int J Nurs Stud. 2018;79:155–64. doi: 10.1016/j.ijnurstu.2018.01.002 29334638

[47] Vandersmissen J, Dewachter I, Cuypers K, Hansen D. The impact of exercise training on the brain and cognition in type 2 diabetes, and its physiological mediators: a systematic review. Sports Med Open. 2025;11(1):42. doi: 10.1186/s40798-025-00836-7 40274715 PMC12022206

[48] Lenze EJ, Voegtle M, Miller JP, Ances BM, Balota DA, Barch D, et al. Effects of mindfulness training and exercise on cognitive function in older adults: a randomized clinical trial. JAMA. 2022;328(22):2218–29. doi: 10.1001/jama.2022.21680 36511926 PMC9856438

[49] Quansah M, David MA, Martins R, El-Omar E, Aliberti SM, Capunzo M, et al. The beneficial effects of lactobacillus strains on gut microbiome in alzheimer’s disease: a systematic review. Healthcare (Basel). 2025;13(1):74. doi: 10.3390/healthcare13010074 39791681 PMC11720007

[50] Aliberti SM, Sacco AM, Belviso I, Romano V, Di Martino A, Russo E, et al. Potential impact of physical activity on measures of well-being and quality of life in people with rare diseases: a nationwide cross-sectional study in Italy. Healthcare (Basel). 2024;12(18):1822. doi: 10.3390/healthcare12181822 39337163 PMC11431722

